# Evidence for a causal role by mouse mammary tumour-like virus in human breast cancer

**DOI:** 10.1038/s41523-019-0136-4

**Published:** 2019-11-07

**Authors:** James S. Lawson, Wendy K. Glenn

**Affiliations:** School of Biotechnology and Biomolecular Science, University of New South Wales, Sydney, NSW Australia

**Keywords:** Breast cancer, Cancer, Oncogenesis

## Abstract

We have reviewed the evidence relevant to mouse mammary tumour viruses (MMTV) and human breast cancer. The prevalence of MMTV- like gene sequences is 15-fold higher in human breast cancer than in normal human breast tissue controls and is present in up to 40% of human breast cancers. MMTV-like gene sequences can be identified in benign breast tissues 1–11 years before the development of positive MMTV-like breast cancer in the same women. The prevalence of MMTV antibodies in sera from women with breast cancer is 5-fold higher than in normal women. MMTV can infect human breast epithelial cells and integrate at random into the human genome located in those cells. MMTV-like gene sequences are present in human milk from normal lactating women and with increased prevalence in milk from women at risk of breast cancer. MMTV-like virus associated human breast cancer has strikingly similar features to MMTV-associated mouse mammary tumours. These features include almost identical nucleotide sequences and structure of the MMTV genome, histology, superantigen expression, MMTV infection of B and T lymphocytes and hormone dependence. MMTV-like gene sequences have also been identified in dogs, cats, monkeys, mice and rats. Saliva has been identified as the most plausible means of transmission from human to human and possibly from dogs to humans. The evidence meets the classic causal criteria. A causal role for MMTV-like viruses in human breast cancer is highly likely.

## Introduction

We have reviewed the evidence relevant to the underlying causes of breast cancer. In our view, there is now sufficient evidence to conclude that a mouse mammary tumour-like virus (MMTV-like) is probably causal in a significant number of breast cancers. The evidence was assessed by using an extended version of the classic Austin Bradford Hill causal criteria.^1^ He established the following criteria—strength of association, consistency, specificity, temporality, biological gradient, plausibility, coherence, experiment and analogy. We have included additional criteria to address more current scientific developments in studies of oncoviruses. These are—the presence of viral genetic material in cancer tissues but rarely in normal tissues, the virus being capable of transforming normal cells into malignant cells, a potential viral oncogenic mechanism and the potential means of viral transmission. There are differences in the importance of each criterion. With respect to viruses and cancer the presence of viral genetic material prior to the development of same virus positive cancer, the strength of association and a significant odds ratio between cancer and normal tissues, are of special importance.

### Background

In 1936, John Bittner discovered a pathogenic agent, which he called a “milk factor’, which could be transmitted by milk from mouse mothers with mammary tumours to their mouse pups, who as adults, later developed mammary tumours.^2^ A decade later, Samuel Graff and colleagues identified viral particles in mouse milk which could cause mammary cancer when injected intraperitoneally into laboratory strains of mice.^3^ These virus particles were later identified as a retroviral RNA virus, which became known as mouse mammary tumour virus.^4^ A period of intensive studies into the biology of MMTV and its possible role in human breast cancer, financed by President Richard Nixon’s “war on cancer”, followed between 1970 and 1985. However, although there were numerous sporadic reports, no definitive links between MMTV and human breast cancer were convincingly identified. As a consequence of this, but also the discovery of human endogenous retroviruses (HERVs), which are similar to currently known exogenous viruses and which many thought accounted for these sporadic reports, interest linking MMTV and breast cancer waned. As a result of the development of sophisticated new techniques such as polymerase chain reaction (PCR), which have enormously increased the sensitivity of detection, there has been renewed interest in the search for a potential role of MMTV in human breast cancer.

### Search strategy and selection criteria

The main source of publications cited in this review is PubMed Central using the search terms breast cancer, mouse mammary tumour virus and mouse mammary tumours—from 1936 to 2019. The publications listed in Table 1, which report the results of case control studies of MMTV in human breast cancer were selected because of the quality of the methods used. Studies which did not investigate normal breast tissues or investigated expression of non-*env* gene sequences or where the primers used were not traceable, were excluded.

**Table 1:** Identification of MMTV sequences in breast cancer and comparison non—cancer breast specimens (case control studies)

Study author year	Location	Breast cancer diagnosis	Breast non-cancer controls	Method	MMTV-positive/total breast cancer specimens	MMTV-positive/total normal or benign breast specimens	Significance for difference MMTV-positive cancer/non-cancer
Mesa-Tejada^76^	US	Invasive	Normal breast	Immunoperoxidase gp52	51/131 39%	0/18 0%	*p* < 0.01
Wang^8^	US	Invasive	Normal breast	PCR	121/314 38.5%	2/107 2%	*p* < 0.01
Etkind^77^	US	Invasive	Normal breast	PCR	27/73 37%	0/35 0%	*p* < 0.01
Melana^78^	US	Invasive	Adjacent normal breast	PCR	32/106 30%	1/106 1%	*p* < 0.01
Melana^79^	Argentina	Invasive		PCR	23/74 31%	1/10 10%	*p* < 0.01
Ford^12^	Australia	DCIS Invasive	Benign breast	PCR	DCIS 5/19 26% IDC 14/26 54%	2/111 2%	*p* = 0.01
Ford^80^	Australia	DCIS Invasive	Normal	PCR	DCIS 2/8 25% IDC 43/136 32%	0/111 0%	*P* < 0.01
Zammarchi^81^	Italy	Invasive	Adjacent normal breast	PCR	15/45 33%	0/8 0%	*p* < 0.01
Hachana^82^	Tunisia	Invasive	Adjacent normal breast	PCR	17/122 14%	0/122 0%	*p* < 0.01
Lawson^64^	Australia	Invasive	Normal cosmetic	In situ PCR	33/74 45%	0/29 0%	*p* < 0.01
Mazzanti^11^	Italy	DCIS Invasive	Normal cosmetic	PCR	DCIS 40/49 82% IDC 7/20 35%	0/20 0%	*p* < 0.01
Glenn^83^	Australia	Invasive	Normal cosmetic	PCR	39/50 78%	13/40 33%	*p* < 0.01
Slaoui^84^	Morocco	Invasive	Adjacent normal breast	PCR	24/42 57%	6/18 33%	*p* < 0.05
Cedro- Tanda^16^	Mexico	DCIS Invasive	Adjacent normal breast	PCR	57/458 12%	72/458 16%	*p* < 0.08
Naushad^85^	Pakistan	Invasive	Normal	PCR	83/250 29%	0/15 0%	*p* < 0.01
Shariatpanahi^86^	Iran	Invasive	Benign	PCR	19/59 32%	3/59 5%	*p* < 0.01
Al Dossary^87^	Saudi Arabia	Invasive	Normal benign	PCR	6/101 5.9%	0/51 0%	*p* < 0.05
Seo^88^	Korea	Invasive	Adjacent normal breast	PCR	12/128 (9.4%)	0/128 (0%)	*p* < 0.01

There are variations in the primer sequences used for PCR in the different studies. This adds to the validity of the identification of MMTV in human breast cancer specimens

*DCIS* ductal carcinoma in situ, *PCR* polymerase chain reaction

## Strength and consistency of association between MMTV and breast cancer

### Identification of MMTV-like gene sequences in human breast cancer

There has been reasonably consistent identification of MMTV-like gene sequences in human breast cancers as compared to the extremely low levels of identification in normal and benign breast tissues. Prior to the widespread use of PCR, MMTV-related sequences in human breast cancer cells were identified by hybridisation techniques.^5^ In early studies Callahan and coworkers were able to identify MMTV on Southern blots hybridized to both a full length MMTV specific probe, as well as to probes specific for the typical structures of a retrovirus, *gag-pol* and *env*, in human normal and breast tumour DNA.^5^ Using hybridisation methods Szakacs & Moscinski^6^ also identified DNA sequences homologous to the entire MMTV provirus using *LTR*- long terminal repeat, *gag*, *pol* and *env* probes in 7 (13%) of 52 human breast cancers.^6^

Until the mid-1990’s, there had been a controversy around the distinction of MMTV from human endogenous retroviruses (HERV) using such hybridization techniques, since these two retrovirus families share a high degree of nucleotide homology in certain regions which might lead to positive signals.^7^

### PCR Studies

Using PCR techniques directed at a 660 bp highly conserved portion of the MMTV-*env* gene with only 16% homology to the prototype HERV-K10 human endogenous retrovirus, Wang et al.^8^ from Beatriz Pogo’s lab, were able to demonstrate MMTV- *env* specific sequences in 38.5% of the 314 breast carcinomas and in 6.9% of the 29 breast fibroadenoma samples, compared to only 1.8% of 107 samples of normal breast reduction mammoplasty tissues.^8^ The specificity of these results was confirmed by hybridization to an MMTV-*env* specific probe, as well as by sequencing of the PCR product from 15 of the positive tumours. In all cases the homology to the MMTV-*env* gene ranged from 95 to 99%, while homology to the HERV-K10-*env* gene was found to be less than 15% and to other viral and human genes was found to be maximally 18%.^9^

**Fig. 1:**
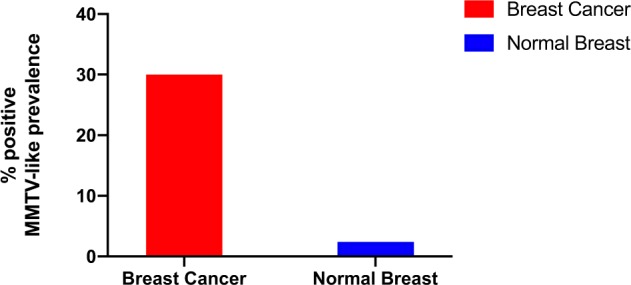
Prevalence of MMTV-like gene sequences in invasive human breast cancers compared to normal human breast tissue controls (based on Table 1)

**Fig. 2:**
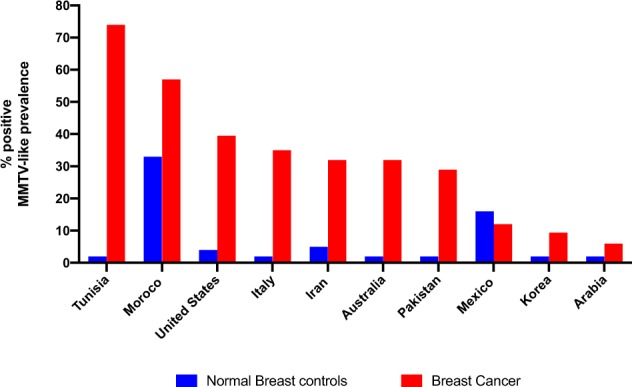
Prevalence of MMTV-like gene sequences in invasive human breast cancers compared to normal human breast tissues in women from 10 selected countries (based on Table 1). The prevalence of MMTV-like sequences in breast cancers varies from a high of 75% in Tunisia to a low of 6% in Saudi Arabia. The prevalence in normal control human breast tissues varies from 33% in Morocco to zero in, Tunisia, Italy, Australia, Pakistan, Korea and Saudi Arabia

The Wang study catalysed a whole series of similar studies using PCR primers and nested primers in an attempt to correlate the presence of the indicative MMTV specific 660 bp *env* sequence with mammary tumorigenesis.^8^ We analysed 20 studies reported in 17 publications in which PCR was used either on total DNA extracted from tumour and normal tissues. Of 15 pure PCR studies, in which tissues from patients with breast cancer were compared to normal non-tumour tissue using primers located within or immediately adjacent to the 660 bp MMTV specific region identified by Wang et al.,11 studies showed a positive correlation between the presence of the indicative MMTV signal and breast tumour tissue at the *p* < 0.01 significance level, two studies, from Morocco and Saudi Arabia, showed the correlation at the *p* < 0.05 significance level, and two studies from Italy and Mexico, showed no significant correlation (Table 1). Based on a meta-analysis of PCR based case control studies by Wang et al. plus later studies, the overall prevalence of MMTV-like gene sequences is 15-fold higher in human breast cancer than in normal human breast tissue controls.^10^ This is shown in Fig. 1. The prevalence of MMTV-like sequences in breast cancers varies from a high of 75% in Tunisia to a low of 6% in Saudi Arabia. The prevalence in normal control human breast tissues varies from 33% in Morocco to zero in, Tunisia, Italy, Australia, Pakistan, Korea and Saudi Arabia. This is shown in Fig. 2.

### Laser microdissection

To further investigate the presence of a signal from a 200 bp subfragment of the highly conserved MMTV *env* region laser microdissection techniques were used to study breast cancer epithelial cells followed by real-time PCR analysis.^11^ MMTV was identified in 40 (82%) of 49 ductal carcinoma in situ specimens and 7 (35%) of 20 invasive ductal carcinoma specimens compared to no identification in 20 normal breast specimens from reduction mammoplasty.

While the outcome of studies of MMTV in human breast cancer are generally consistent, several research groups have experienced difficulties when using PCR techniques for the identification of MMTV gene sequences. This is illustrated by the experience of Park et al. who could not identify MMTV gene sequences in breast cancers in Australian women despite their previous identification by Ford et al.^12,13^ Pogo et al. demonstrated by laboratory experiments that the methods used by Park et al. were inadequate.^14^

## Contamination

Perzova et al. have challenged the validity of the identification of MMTV gene sequences in human breast cancers.^15^ They argue that many of the findings outlined above and which are based on PCR technology, are due to contamination by MMTV with mice and other rodents as the original source. There is laboratory based evidence, which demonstrates that this view is incorrect. Potentially contaminating rodent DNA can be identified by testing PCR products for the presence of murine mitochondrial genomic DNA plus the detection of murine intracisternal A particle long terminal repeats. These techniques have been used to exclude contamination by rodent DNA in five independent laboratories.^16–20^

### Whole-genome sequencing

There are difficulties with the use of whole-genome sequencing for the identification of MMTV in human breast cancer. MMTV sequences were identified in only three of over 800 breast cancer specimens from The Cancer Genome Atlas (TCGA) as compared to the frequent identification by PCR techniques of MMTV sequences in over 40% of breast cancers.^21^ This low identification of MMTV by whole-genome sequencing is possibly due to its limited integration into the host human genome. There are similar experiences with respect to other retroviruses, including bovine leukemia virus and human immunodeficiency virus.

Lehrer and Rheinstein have noted that MMTV pol, ENV and other genes are present in breast tumours and normal breast tissues.^22^ They did not identify the MMTV *env* gene in the human genome or the genomes of other primates. They suggest this finding indicates that the MMTV ENV sequences that were identified in breast tumours and normal breast tissues were from an infection.

### Serology

The prevalence of MMTV antibodies in the serum of women with breast cancer is consistently five-fold higher than in the sera of women with benign breast conditions or in normal women (Table 2). Different methods have been used to measure the specificity of the antibodies in these studies. Regardless of the methods the outcomes are similar with the exception of Kovarik and Goedert et al.^23,24^ Kovarik et al.^23^ identified MMTV antibodies in 2 (3%) of 60 serum samples from women with breast cancer compared to 0 (0%) of normal controls.^23^ Goerdert et al. did not identify any MMTV antibodies in the sera of 92 women with breast cancer.^24^ The outcomes of the Goedert et al. study may not be valid because the sera used was collected 10–15 years before the study and frozen several times. Repeated thawing and freezing degrades antibody proteins.^25^ In addition MMTV gp52 was not identified in either the positive controls or the sera from the patients with breast cancer in the Goedert et al. study.

**Table 2:** Mouse mammary tumour virus human breast cancer serology

Study Year	Location	Diagnosis breast cancer	Control women. Diagnosis	Method to identify MMTV in blood sera	MMTV-positive/total breast cancer	MMTV-positive/normal women	Statistical significance Breast cancer/normal women
Muller^89^	Germany	Breast cancer	Fibrocystic	Immuno fluorescence	75/228 33%	11/95 12%	*p* < 0.01
Ogawa^90^	Japan	Breast cancer	Fibroadenoma	Immuno fluorescence	26/43 60%	4/37 11%	*p* < 0.01
Mehta^91^	India	Breast cancer		Immuno fluorescence	26/34 76%	0/10 0%	*p* < 0.01
Witkin^92^	US	Breast cancer	Benign	Virolytic assay	11/65 17%	2/60 3%	*p* < 0.01
Imai^93^	Japan	Breast cancer	Benign	Immuno fluorescence	49/89 55.1%	18/68 27%	*p* < 0.01
Witkin^94^	US	Breast cancer	Benign	ELISA gp52, gp34	14/54 26%	5/63 8%	*p* < 0.08
Day^61^	US, India, China, Africa	Breast cancer	Normal women	ELISA	US 27/145 19% India 20/53 38% Kenya 7/26 27% China 1/22 5%	US 1/36 3%	*p* < 0.02
Nagayoshi^95^	Japan	Breast cancer	Other cancers	Hemaglutination	34/96 36%	3/59 5%	*p* < 0.01
Tomana^96^	US	Breast cancer	Benign	Immuno fluorescence	56/137 41%	2/56 4%	*p* < 0.01
Zotter^97^	Germany	Breast cancer	Benign fibrocystic	Radio Immuno precipitation	84/367 23%	11/184 6%	p < 0.01
Holder^25^	US	IDC	Other cancers	Viral agglutination	41/52 79%	2/18 11%	*p* < 0.01
Litvinov^98^	Russia	Breast cancer	Other cancers	Radio Immuno assay	51/92 55%	3/94 3%	*p* < 0.01
Chattopadhyah^99^	India	Breast cancer	Normal women	Hemaglutination	14/14 100%	0/13 0%	*p* < 0.01
Kovarik^23^	Czechoslovakia	Breast cancer	Normal women	Immunoblotting	2/60 (3%)	0/60 (0%)	*p* < 0.16

These data indicate the response of the immune system to MMTV as a pathogen. There is a consistently higher immune response to MMTV in women with breast cancers as compared to normal women

*IDC* invasive ductal carcinoma

Laboratory workers exposed to MMTV have showed serological responses to the MMTV *env* (gp52 and gp34), and *gag* (p12, p18, p28) proteins.^26^

## Epidemiology

MMTV-like sequences have been identified in 30 and 40% of invasive breast cancers in Western countries and between 10 and 20% in Asian countries. The differences may be due to the geographical differences in the prevalence of MMTV in mice-, which may influence the prevalence of MMTV in humans.^27^ It has been proposed that the higher the prevalence of one species of house mouse, *Mus domesticus*, the higher the prevalence of human breast cancer.^27^

There have not been any studies of associations between MMTV-like positive breast cancers and immunophenotypes. While there are correlations between MMTV-like positive human invasive breast cancers and histological characteristics similar to MMTV-positive mouse mammary tumors, there do not appear to be any associations with histological types.^21^

Because breast cancer is more prevalent among women of higher socio-economic status, Lehrer and Rheinstein have hypothesised this may be due to exposure to infection by MMTV from mice during early life among girls of low socio-economic status and hence gaining early immunity.^28^

## MMTV prevalence in BRCA hereditary compared to sporadic breast cancers

In an intriguing new study, Naccarato et al. report the identification of MMTV-like gene sequences (MMTV) in 17 (30.3%) of 56 sporadic human breast cancers as compared to 2 (4.2%) of 47 hereditary human breast cancers.^20^ The difference is statistically significant (*p* = 0.001). As hereditary breast cancers are known to be associated with BRCA gene mutations and are not associated with oncogenic viruses, the implication of these observations is that MMTV has a likely role in sporadic breast cancers.

## Transformation

**Fig. 3:**
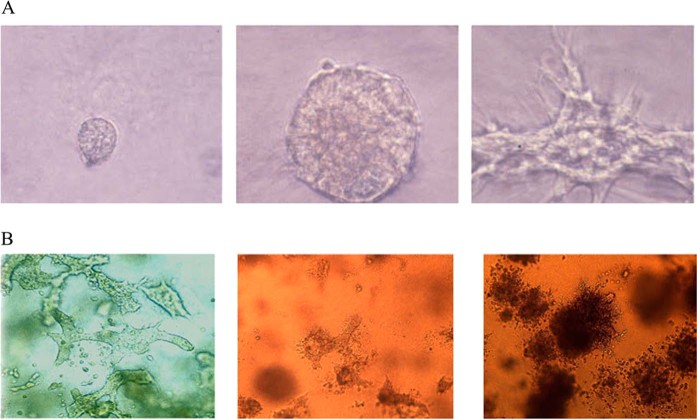
Mouse mammary tumour virus envelope proteins transform normal human mammary epithelial cells into invasive breast cancer cells. The images demonstrate transformation from **a** normal mouse mammary cells to invasive cancer cells **b** normal human breast ductal cells following experimental exposure to MMTV. Figure used with permission from Rockefeller University Press under licence number 4672720172161. The Figure is based on two separate figures in Katz et al.^29^

Proteins expressed by the MMTV envelope gene are capable of malignantly transforming normal human breast epithelial cells.^29^ This shown in Fig. 3.^29^ MMTV envelope protein p14 overexpression can function in an oncogenic capacity.^30^ MMTV encoded proteins (such as Rem, Sag, Naf) or as yet uncharacterised proteins analogous to those of other complex retroviruses such as Tax may also have a role in breast cancer.

## Specificity

MMTV-like virus is not specific for breast cancer. MMTV-like virus has been identified in a number of human cancers including breast, lymphoma, prostate, liver and endometrial liver, endometrial, ovary, prostate skin and lung cancers and also in biliary cirrhosis.^31,32^ Apart from breast cancer, there is no evidence available to determine whether MMTV is causal in these cancers. MMTV in mice is also associated with mammary, lymphomas, salivary gland tumours and with renal adenocarcinoma.^33^

## Temporality

### MMTV infection and subsequent breast cancer time sequence

MMTV has been identified by both PCR and immunohistochemistry techniques in normal and benign human breast tissues before the development of MMTV-positive breast cancer 1–11 years later in the same women.^17,34^

## Biological gradient

As human breast cancer progresses, the MMTV viral load increases but it falls in late stage invasive breast cancer.^11,12^ The lessened identification of MMTV in advanced breast cancer may be due to the breakdown of cell physiology.

## Plausibility—identification of MMTV in other mammals

MMTV is the proven cause of mammary cancers in wild (feral) mice.^33^ Gene sequences with high homology to MMTV have been identified in rhesus monkeys and cats although the tissues studied were not mammary tumours.^35^ MMTV-like gene sequences have been identified in 20% of mammary tumours in dogs and 33% of mammary tumours in cats.^36^ Civita et al.^19^ have identified MMTV gene sequences in cat mammary tumours but not in dog mammary tumours.^19^ The findings by Civita et al., based on PCR, were confirmed by immunohistochemistry using MMTV p14 antibodies.^19^ Accordingly, it is plausible that MMTV may also cause mammary tumours in humans.

## Experimental evidence

### Capacity of MMTV to infect human breast epithelial cells

**Fig. 4:**
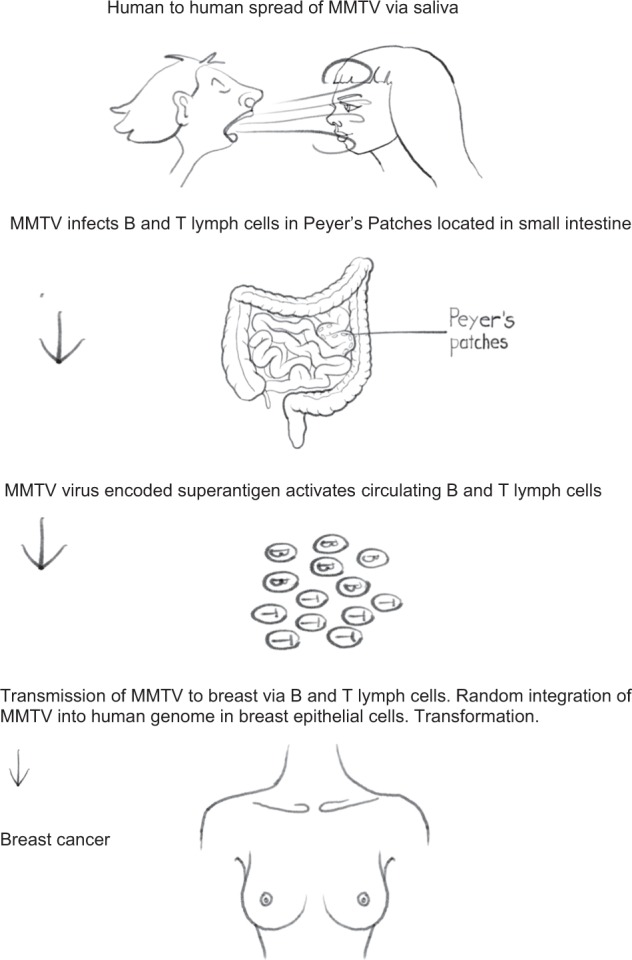
Mouse mammary tumour virus infects human breast epithelial cells. The detection of MMTV in the cells is by MMTV- enhanced green fluorescent protein (based on experiments conducted by Konstantoulas and Indik^38^)

MMTVs can infect human breast cells in culture.^37,38^ This shown in Fig. 4. Faschinger et al. demonstrated that when MMTVs infect human mammary epithelial cells they randomly integrate their genomic information into the human genome of the infected cell.^38^ Following integration of MMTV into the human genome of the infected mammary epithelial cells the flanking sequences are of human and not mouse origin. This is an indication of an exogenous infection rather than contamination.^39^ Integration of MMTV into the human genome appears to be random and in multiple locations. Faschinger et al. also demonstrated there is a similar pattern of random MMTV integration into the mouse genome.^39^

Overall, this evidence indicates an infective transmission of MMTV in humans.

## Analogy

### Analogy between MMTV-associated mouse mammary tumours and MMTV-associated human breast cancer

**Fig. 5:**
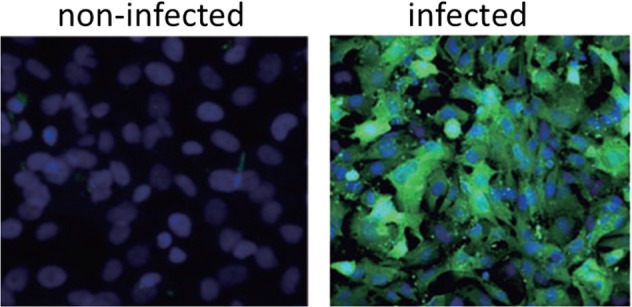
Possible etiopathogenesis of MMTV-associated human breast cancer. The evidence in support of this possible etiopathogenesis for MMTV in human breast cancer is not as robust as that for MMTV in mice but is substantial.^18,29,37,38,42,51^

The biology of MMTV in mice is very similar to the apparent biology of MMTV in humans.^33^ In mice MMTV is spread by the milk of infected mouse mothers and is acquired by suckling pups. MMTV is also present in the salivary glands of mice.^33^ Ingested MMTV enters T and B cells located in Peyer’s patches of the gastrointestinal tract of infected mouse pups. This spread of MMTV infection requires activation of T and B lymphocytes by the viral superantigen (SAg). SAg activation allows the virus to amplify in lymphocytes prior to transmission of the virus to mammary epithelial cells. Ultimately the virus is transported by infected lymphocytes to the mammary glands where oncogenic transformation takes place. In humans, MMTV is present in saliva and human milk and after ingestion infects T and B lymphocytes located in the Peyers patches in the intestine, which are activated by SAg and randomly infects and integrates into the human genome located in normal breast epithelial cells.^18,40–42^ The possible MMTV etiopathogenesis in human breast cancer is shown in Fig. 5.

The evidence related to MMTV-associated human breast cancer and MMTV-associated mouse mammary tumours, is remarkably similar.

1. MMTV has been identified globally in human breast cancer and mouse mammary tumours.^10,27^ The highest incidence of breast cancer occurs in locations where *Mus domesticus* is the resident native or introduced species of house mouse.^27^ MMTV prevalence is higher in *Mus domesticus* mice than in other mice strains.
2. Virus particles have been identified in human milk from women with breast cancers.^43^ These particles in human milk are physically similar to MMTV particles identified in mice.^43^ Virus particles from human breast cancer cells contain MMTV sequences.^9^ MMTV gene sequences have been identified in human milk from normal lactating Australian and US women.^40,41^
3. The nucleotide sequences and structure of MMTV-like viral sequences that have been identified in human breast cancer tissues are virtually identical to MMTV sequences identified in mouse mammary tumours.^9,44^
4. Abnormal cancer-related gene expression is similar in mouse mammary tumours and human breast cancers.^45,46^ Klein et al. identified the same 63 genes, which were differentially expressed in over 75% of both human breast cancers and mouse mammary tumours.^46^
5. MMTV-associated envelope and capsid protein expression is similar in both mouse mammary tumours and human breast cancers.^30,47,48^
6. Superantigen (SAg) expression. MMTV SAgs play an essential role in MMTV-associated mouse mammary tumours.^49^ MMTV-infected B lymphocytes and dendritic cells lead to the expression of virus SAgs, which in turn stimulate T-cells to produce cytokines that encourage the proliferation of infected B lymphocytes thereby forming a reservoir of infected cells. MMTV is then conducted throughout the body by these lymphocytes, which facilitate the entry of MMTV into its target organ (the mammary glands or other organs). MMTV SAg is highly expressed in MMTV-associated human breast cancer and may have a similar role in humans as it does in the mouse. Human T cells respond to MMTV SAg.^50,51^
7. MMTV infects intestinal T and B lymphocytes and randomly integrates into both the mouse and human genome.^33,38,39^ Similarly, MMTV-infected human lymphocytes can be found in women with MMTV-positive breast cancer.^52–54^ In immunocompromised mice injected with wild-type MMTV, there is no infection of peripheral lymphocytes or virus spread to the mammary glands.^50^ The risk of breast cancer is reduced by 44% in immunocompromised human patients.^55^ This is in contrast to immunocompromised patients with other cancers whose risk of cervical and other cancers increases markedly.^56^ These observations are compatible with the notion that an intact immune system is required for the transmission of MMTV by lymphocytes in both mice and humans.
8. MMTV-associated tumour histology appears to be similar in both mouse mammary tumours and MMTV-positive human breast cancers.^21,57^ It is of interest that this similarity mainly applies to mouse mammary tumours in mice who were infected with MMTV during the neonatal period (for more detail see the section on transmission below^58^). However, the MMTV-associated histology is not necessarily specific to human breast cancer as there are similarities to other cancers such as squamous cell skin cancers. A recent study has shed some clarity to this issue. Based on a study of genes and histology, Hollern et al. have shown that many features of murine tumour histologies are conserved in both mouse and humans and in several different cancer types.^59^
9. Antibodies to the MMTV surface envelope gp52 are present in the serum and mammary glands of feral mice.^60^ Antibodies to MMTV gp52 have been identified in the serum of 2.8% of normal US women.^61^ However, the repeated identification of MMTV gp52 antibody in human breast cancers has yet to be confirmed by modern methods in humans.^24^
10. Hormones. Mammary tumours in some strains of mice are hormone dependent.^62^ In humans, MMTV hormonal response elements in breast cancers appear to promote cell growth as they do in mice.^63^
11. Wnt-1 is an oncogene, which is highly expressed in MMTV-associated mouse mammary tumours.^33^ In humans, there is suggestive evidence that the influence of MMTV on human breast tissues cells leads to abnormally high Wnt-1 expression.^64^

MMTV has been identified in mammary tissues in feral mice and in normal human breast tissues prior to the development of MMTV-positive mouse mammary tumours and MMTV-positive human breast cancers.^17,34^

## MMTV transmission

The most plausible means of transmission of MMTV in humans is via human saliva.^18^ MMTV gene sequences are present in saliva in 27% of normal children, 11% of normal adults and 57% of women with breast cancer.^18^ MMTV-like gene sequences have been identified in human parotid glands—the source of saliva.^18^ MMTV can infect adult mice via nasal lymphoid tissue. Humans have well developed lymphatic structures in the mouth and nose (tonsils and adenoids), which are possible entry points for MMTV.

The father, mother and daughter of a family living together, all developed MMTV-associated breast cancer.^65^ The MMTV *env* gene sequences identified in each family member was at least 98% homologous to the MMTV *env* sequences found in laboratory mouse strains. This observation supports the evidence that transmission of MMTV is likely to be by saliva.

The prevalence of MMTV in human milk is significantly higher among women who are at greater than normal risk of breast cancer.^41^ This suggests that human milk is a possible means of MMTV transmission.

MMTV gene sequences have been identified in mammary tumours in dogs and cats.^19,35,36^ Women with companion dogs are at twice the expected risk of breast cancer, which suggests MMTV could be transmitted in dog saliva to humans.^66^

In many countries it is permissible for 1% in weight of cereals to consist of mouse or rat faecal material.^67^ Because MMTV is endemic in many rodent populations, transmission by consumption of uncooked cereals and other foods is possible.

Overall this evidence suggests that while human to human saliva is the most likely means of MMTV-like transmission. Additional means of transmission are also possible.

## MMTV oncogenic mechanisms in human breast cancer

The underlying causal mechanisms by which MMTV may cause human breast cancer are far from clear. With respect to laboratory mice it has been demonstrated that integration of MMTV proviral DNA into the mouse genome near one or more of the proto-oncogenes such as Wnt-1 and Fgf, is associated with the development of mouse mammary tumours. However, more recent studies have indicated that MMTV oncogenesis in mouse mammary tumours is more complex than insertional oncogenesis. MMTV envelope genes encode immunoreceptor tyrosine-based activation motif (ITAM) containing proteins, which are capable of malignantly transforming mouse mammary epithelial cells.^29,47^

The relatively low levels of MMTV-like gene sequences that are detected in human breast cancers suggest that the virus is affecting oncogenesis by mechanisms in addition to MMTV insertion. Additional mechanisms by which MMTVs can contribute to human breast oncogenesis include (i) proteins expressed by the MMTV envelope gene and which are capable of malignantly transforming normal human breast epithelial cells,^29^ (ii) MMTV envelope protein p14 overexpression can function in an oncogenic capacity^30^ (iii) MMTV encoded proteins (such as Rem, Sag, Naf) or as yet uncharacterised proteins analogous to those of other complex retroviruses such as Tax may also have a role in breast cancer. The envelope proteins of Jaagsiekte Sheep Retrovirus (JSRV) which like MMTV is a beta retrovirus, can directly transform cells and offers a precedent for the ability of viral envelope proteins to malignantly transform cells.^68^ There is also the intriguing possibility that MMTV and human endogenous retroviruses may interact and thus also play a role.^30^

The enzyme APOBEC3B is an additional potential oncogenic mechanism. APOBEC3B is an enzyme, which inhibits retrovirus replication. In mice, APOBEC3 has been shown to inhibit MMTV infections and viral replication and to inhibit milk borne MMTV virions.^69^ In humans, inactivating mutations and deletions in APOBEC3B appear to play a role in breast cancer development.^70^ A deletion polymorphism in the APOBEC3B gene cluster on chromosome 22 is associated with elevated breast cancer risk and a specific APOBEC mutation pattern has been described in a number of cancers, including breast cancer.^70^ This mutation pattern is linked to APOBEC3B expression specifically in breast cancer and leads to DNA damage that could select TP53 inactivation, thereby resulting in tumour heterogeneity.^69^ Human papilloma viruses have been shown to alter the expression of APOBEC3B, which may reduce its protective effects against MMTV.^71,72^ It has also been shown that there is a significant increase in APOBEC-mediated mutagenesis in HER+/HER2 metastatic breast tumours as compared to early stage primary breast cancer. Taken together, these studies suggest that the APOBEC family and particularly APOBEC3B, may play a key role in the early stages of breast cancer induction.

## Multiple Oncogenic viruses and breast cancer

There is substantial evidence that additional oncogenic viruses may have roles in breast cancer. These are bovine leukemia virus, high risk human papilloma viruses and Epstein Barr virus (EBV).^73^ As discussed above the role of these viruses is complex and may be indirect. This is the same way that human immunodeficiency virus (HIV) has an indirect role in Kaposi’s sarcoma.^73^ In addition, there may be collaboration between oncoviruses in breast cancer. For example there is evidence that EBV and human papilloma viruses may interact and lead to increased cell proliferation.^73,74^

## Prevention

Three very different methods using experimental mice, have been used to develop protection against MMTV induced mouse mammary tumours. The first method has been the use of formalin inactivated mouse mammary tumour viruses as vaccines. The second method has been the use of MMTV based peptides and proteins. The third method is the use of vaccines prepared from cells of mouse mammary tumours. Each of these methods has been successful in reducing either MMTV infections in mice or mouse mammary tumour development. Recently the Hochman group working in Israel, together with the Bevilacqua group in Italy, have identified specific MMTV envelope proteins, which can potentially be used for both prevention and treatment of MMTV-associated human breast and other cancers.^75^

## Conclusions

The evidence is sufficiently detailed and comprehensive to conclude:

(i) DNA gene sequences, which are almost identical to mouse mammary tumour virus (MMTV) sequences are present in up to 40% of human breast cancers.
(ii) MMTV-like gene sequences are up to 15-fold more prevalent in human breast cancers as compared to normal and benign breast tissues in the same populations of women.
(iii) MMTV-like gene sequences and MMTV p14 proteins can be identified in normal and benign breast tissues 1–11 years before the development of MMTV-like positive breast cancer in the same women.
(iv) MMTV can infect human mammary epithelial cells and integrate at random into the human genome located in those cells.
(v) The prevalence of MMTV antibodies in sera from women with breast cancer is five-fold higher than in normal women.
(vi) Proteins expressed by the MMTV envelope gene can malignantly transform normal human breast epithelial cells.
(vii) Saliva has been identified as the most plausible means of transmission from human to human and possibly from dogs to humans.
(viii) MMTV-like gene sequences are present in human milk from normal lactating women and with increased prevalence in milk from women at risk of breast cancer.
(ix) MMTV-like gene sequences have also been identified in dogs, cats, monkeys and other mammals including mice and rats.
(x) There is a striking similarity in the features of MMTV-like virus associated human breast cancer compared to MMTV-associated mouse mammary tumours. The nucleotide sequences and structure of the MMTV-like and MMTV viruses are almost identical. Abnormal gene expression, possibly histology, superantigen expression, MMTV infection of B and T lymphocytes and hormone dependence are all similar in both MMTV-like positive human breast cancers and MMTV-associated mouse mammary tumours.

The evidence with respect to MMTV being responsible for a subset of approximately 30–40% of human breast cancers in Western countries and 10–20% in Asian countries is compelling. An exception is the lack of detailed information about causal mechanisms.

A role for MMTV-like viruses in human breast cancer is highly likely.
